# Effect of CaS Nanostructures in the Proliferation of Human Breast Cancer and Benign Cells In Vitro

**DOI:** 10.3390/app122010494

**Published:** 2022-10-18

**Authors:** Daniel Rivera Vazquez, Kevin Munoz Forti, Maria M. Figueroa Rosado, Pura I. Gutierrez Mirabal, Edu Suarez-Martinez, Miguel E. Castro-Rosario

**Affiliations:** 1 School of Biological and Physical Sciences, Northwestern State University, Natchitoches, LA 71457, USA; 2 Department of Chemistry, The University of Puerto Rico at Mayaguez, Mayaguez 00680, Puerto Rico, USA; 3 Department of Biology, The University of Puerto Rico at Ponce, Ponce 00716, Puerto Rico, USA

**Keywords:** nanotechnology, cancer, calcium, sulfide, calcium sulfide, cell cycle, apoptosis

## Abstract

We report on the effect of naked CaS nanostructures on the proliferation of carcinoma cancer cells and normal fibroblasts in vitro. The CaS nanostructures were prepared via the microwave-mediated decomposition of dimethyl sulfoxide (DMSO) in the presence of calcium acetate $\text{Ca}{\left({\text{CH}}_{3}{\text{CO}}_{2}\right)}_{2}$. Light scattering measurements revealed that dispersions contain CaS nanostructures in the size range of a few Å to about 1 nanometer, and are formed when DMSO is decomposed in the presence of $\text{Ca}{\left({\text{CH}}_{3}{\text{CO}}_{2}\right)}_{2}$. Theoretical calculations at the DFT/B3LYP/DGDZVP level of theory on ${\left(CaS\right)}_{n}$ clusters (n=1,2,3, and 4) are consistent with clusters in this size range. The absorption spectra of the CaS nanostructures are dominated by strong bands in the UV, as well as weaker absorption bands in the visible. We found that a single dose of CaS nanoclusters smaller than 0.8 nm in diameter does not affect the survival and growth rate of normal fibroblasts and inhibits the proliferation rate of carcinoma cells in vitro. Larger CaS nanostructures, approximately (1.1 ± 0.2) nm in diameter, have a similar effect on carcinoma cell proliferation and survival rate. The CaS nanoclusters have little effect on the normal fibroblast cell cycle. Human carcinoma cells treated with CaS nanocluster dispersion exhibited a decreased ability to properly enter the cell cycle, marked by a decrease in cell concentration in the G0/G1 phase in the first 24 h and an increase in cells held in the SubG1 and G0/G1 phases up to 72 h post-treatment. Apoptosis and necrotic channels were found to play significant roles in the death of human carcinoma exposed to the CaS nanoclusters. In contrast, any effect on normal fibroblasts appeared to be short-lived and non-detrimental. The interaction of CaS with several functional groups was further investigated using theoretical calculations. CaS is predicted to interact with thiol (R-SH), hydroxide (R−OH), amino ($\text{R}-{\text{NH}}_{2}$), carboxylic acid (R−COOH), ammonium (${\text{R-NH}}_{3}^{+}$), and carboxylate (${\text{R-COO}}^{-}$) functional groups. None of these interactions are predicted to result in the dissociation of CaS. Thermodynamic considerations, on the other hand, are consistent with the dissociation of CaS into ${\text{Ca}}^{2+}$ ions and ${\text{H}}_{2}\text{S}$ in acidic media, both of which are known to cause apoptosis or cell death. Passive uptake and extracellular pH values of carcinoma cells are proposed to result in the observed selectivity of CaS to inhibit cancer cell proliferation with no significant effect on normal fibroblast cells. The results encourage further research with other cell lines in vitro as well as in vivo to translate this nanotechnology into clinical use.

## 1. Introduction

The use of structured nanomaterials promises to have a positive impact on the development of cancer treatments as well as detection and imaging [1–5]. Materials for cancer therapy require careful consideration regarding nanostructure shape and dimensions, surface charge, chemical composition, and hydrophobicity, as well as ligand size, orientation, density, and charge [5]. These factors are important and relevant to the toxicology, specificity, and clearance of the therapeutic agent in the human body. Structures with sizes that range from a few Å up to 5 nm, for instance, are small enough to enter the smallest blood vessels in humans, including a new vasculature developed as part of metastatic processes. Structures in that size range are also compatible with renal clearance (threshold value of ~5.5 nm) [6] and can penetrate pores in cells in the size range of 2 to 8 nm [7].

Nanotechnologies employed in cancer diagnosis and treatment generally consist of a core and a chemical modifier on the surface of the structure that adds molecular recognition and biocompatibility. These nanotechnologies include nanoparticles, polymeric micelles and liposomes, dendrimers, nanocantilevers, carbon nanotubes, and quantum dots. The most common applications for these materials vary, with liposomes, dendrimers, and polymeric micelles usually being employed as encapsulating agents and drug delivery vehicles, nanoparticles and quantum dots as therapeutic agents, and functionalized carbon nanotubes as platforms for both therapeutic agents and drug delivery systems. Functionalized nanocantilevers have tremendous potential for early cancer biomarker detection—including antibodies and antigen detection in vitro—at the single molecule level and can impact early detection of the condition.

Current developments in nanotechnology that rely on functionalization to target specific cells and/or tissue usually result in structures with hydrodynamic radios that are too large to fulfill the size requirement to enter the small vasculature developed in tumor angiogenesis and are complex organic or organometallic structures with significant metabolic pathways causing several secondary effects. Such structures, while increasing the residence time of a target drug in the human body, can present a clearance problem by the kidneys and may have limited direct action on cancer cells trapped in small human vasculature associated with the metastatic process and may bring new metabolic processes that limit the amount of intake by humans. In these regards, naked CaS nanostructures and clusters have potential applications as target cell cancer and metastasis therapy agents.

CaS is a homeopathic remedy known as *Hepar sulphuris*. It is used as a remedy in alternative medicine to treat colds, coughs, sore throats, croup, abscesses, earaches, inflamed cuts and wounds, asthma, arthritis, emphysema, herpes, constipation, conjunctivitis, *Candida albicans* infections, syphilis, sinusitis, and skin infections [8]. However, the effect of CaS nanostructures on the growth and survival rate of cancer cells is unknown. This is surprising given the fact that it finds wide use as an alternative medicine for the symptoms listed above. The importance of rigorous scientific studies to establish the action of homeopathic remedies to fight cancer—as well as other diseases—has been highlighted recently. Such studies will facilitate easy and reliable medical advice from pseudoscience that is broadly available to the public [9].

Compared to many popular nanomaterials—including noble metals and magnetic nanoparticles—calcium and sulfur-based nanostructures are promising biocompatible inorganic materials. Calcium is an abundant mineral in the human body. It plays major roles in both macroscopic biological functions such as bone remodeling, and biological functions at the molecular level such as intracellular and extracellular communication, protein structures, and mitochondrial metabolism. Sulfides, on the other hand, are present in the human body mostly in organic compounds such as proteins and genetic material, and it is now postulated as a novel neuromodulator/transmitter with important physiological properties in the form of ${\text{H}}_{2}\text{S}$ [10–12]. CaS nanostructures also have the potential to play an important role as cadmium-free nanostructures with applications in bioimaging [13–19] and in vivo labeling [20–22] and sensing [23–27].

We report on the effect of naked CaS nanostructures on the proliferation and survival rate of carcinoma cancer cells (ATCC CRL-2124) and normal fibroblasts (CRL-2522, ATCC) in vitro. Passive uptake and extracellular pH of carcinoma cells are proposed to result in the selectivity of CaS to inhibit human breast cancer cell proliferation with no effect on normal fibroblast cells.

## 2. Materials and Methods

### 2.1. Synthesis of CaS Clusters and Nanostructures: Microwave Decomposition of Dimethyl Sulfoxide in the Presence of Calcium Ions

CaS was prepared from the microwave-mediated decomposition of DMSO in the presence of $\text{Ca}{\left({\text{CH}}_{3}{\text{CO}}_{2}\right)}_{2}$. Calcium acetate (Fisher Scientific, CAS no. 62–54-4) and DMSO (Acros Organics 99.7%) were used without further purification. The desired amount of $\text{Ca}{\left({\text{CH}}_{3}{\text{CO}}_{2}\right)}_{2}$ employed was measured using a Metler Toledo AT 20 microbalance. The $\text{Ca}{\left({\text{CH}}_{3}{\text{CO}}_{2}\right)}_{2}$ was dissolved in DMSO and sonicated for 10 min. The microwave synthesis was performed by exposing this solution to microwave radiation in cycles of 5-s “on” and 15-s “off”. This process was repeated for up to 15 intervals of exposure to microwave radiation in the sample [28,29]. The resulting solution was filtered several times using a P5 Fisher filter paper prior to the measurements reported here.

### 2.2. Characterization of CaS Nanostructures

Experimental determination of the UV-Vis absorption spectroscopy measurements was performed using a PC 2000 Ocean Optics UV-Vis spectrometer. Fluorescence spectroscopy measurements were performed in a homemade setup. The 405 nm line from a Wicked Laser E3 laser was employed as the excitation source. Emission measurements were performed in a 1 mL quartz cuvette with the Ocean Optics PC 2000 spectrometer. A research-grade fiber optic was used to couple the spectrometer to the sample holder containing the cuvette. All emission measurements were performed at an angle of 90° with respect to the incident laser line. Light scattering measurements were performed in a calibrated dynamic 1000 D scattering model.

### 2.3. Cell Culturing

A human carcinoma fibroblast cell line CCD-1097Sk (ATCC^®^ CRL-2124^™^) from a 31-year-old Caucasian and a non-malignant healthy fibroblast cell line CCD-1074Sk (ATCC^®^ CRL-2090) from a 41-year-old Caucasian female were used for our cell-based analysis. Both were cultured in their respective base media with 10% *v*/*v* fetal bovine serum and a 1% *v*/*v* streptomycin/penicillin cocktail. All cell culture experiments were performed in a clean room under biosafety level 2 conditions. The cells were grown using T-175 cell flasks incubated at 5% CO_2_, 37 °C, and 95% humidity. Microscope images were taken using an inverted microscope and recorded using a 10MP MU1000 digital camera (Amscope, Irvine, CA, USA). Image-based cytometry was performed using a Cellometer K2 (Nexcelom Bioscience LLC., Lawrence, MA, USA) and raw data were analyzed using FCS Express 4 Flow Cytometry (De Novo Software, Glendale, CA, USA). Kits used for image-based cytometry were purchased from Nexcelom Bioscience LLC. (Lawrence, MA, USA) unless stated otherwise.

### 2.4. Treatment of Cell Cultures with CaS Clusters and/or Nanostructures

CaS clusters and nanostructure treatments have a calcium-to-DMSO ratio of 4 and 6 × 10^−4^, respectively. Since the number of clusters or particles employed is unknown, we used the total initial mol of calcium, corrected for subsequent dilution effects, to report an upper limit estimate of the amount of CaS. Based on the initial mass of the calcium acetate employed and assuming 100% conversion into CaS clusters or nanostructures, we estimated a calcium content of the order of 3.8 to 5.7 × 10^−8^ mol of calcium per dose of clusters or nanoclusters in the doses. The CaS clusters or nanostructures in DMSO were mixed with the media so that the total DMSO content was smaller than 2%. DMSO (2%) was used as vehicle control. Negative controls were growth media, unless stated otherwise.

### 2.5. Trypan Blue Viability Assays

Flasks were washed once with 10 mL of Dulbecco’s phosphate-buffered saline 1x (DPBS). A total of 10 mL of Trypsin-Versene (EDTA) was added to detach cells from the flask and left for 8 min. The flasks were observed under a light microscope to verify cells’ detachment. A total of 20 mL of media was added to neutralize the trypsin. The mixture was transferred to 50 mL centrifuge tubes and centrifuged for 11 min at 1400 rpm at 4 °C. Supernatants were stored for future analyses and cell pellets were re-suspended in 1 mL of culture media. We mixed 20 μL of 0.2% Trypan Blue Stain with 20 μL of cell suspension and 20 μL of this mixture (Trypan Blue Stain + cell suspension) was added to the cell counting chamber. These data were used to calculate the corresponding concentrations of cells for Annexin V and PI ell Cycle analysis.

### 2.6. Annexin-V FITC Apoptosis Assay

Cell concentration and the percentage of viability were determined using trypan blue staining. The percentage of apoptotic cells was determined following the manufacturer’s recommendations. In summary, we added 50 μL of a cell suspension with a concentration of $2\times 10^{6}\frac{\text{cells}}{\text{mL}}$ in microcentrifuge tubes and centrifuged at 1800 rpm for 5 min. Media were aspirated and the cells were re-suspended in 40 μL of Annexin V Binding Buffe. Then, mixed ten (10) times with a micropipette followed by the addition of 5 μL of Annexin V-FITC and 5 μL of PI solution. The cells were incubated for 15 min at 25 °C in the dark. Then, 250 μL of PBS 1X was added to the sample and spun down at 1400 rpm for 5 min. The media were discarded, and the cells were re-suspended in 50 μL of Annexin V Binding Buffer. Fluorescence was quantified using image cytometry on a Cellometer K2. Procedures and analysis were conducted in accordance with manufacturer’s recommended procedures.

### 2.7. Cell Cycle Assay

A suspension of cells with an initial concentration of $1\times 10^{6}\frac{\text{cells}}{\text{mL}}$ was spun down at 1400 rpm for 5 min and re-suspended in 100 μL of PBS 1X to obtain a concentration of approximately $10\times 10^{6}\frac{\text{cells}}{\text{mL}}$. Then, 500 μL of ice-cold 200 proof ethanol was gradually added and mixed 10 times with a micropipette, followed by an ice bath for 15 min. Cells were centrifuged at 1400 rpm for 8 min and the ethanol/PBS supernatant was removed and cells were re-suspended in 150 μL of Propidium Iodine (PI) staining solution. We incubated the samples on a heating block at 37 °C for 40 min in the dark and then spun them down at 1400 rpm for 8 min. The staining solution was carefully removed, and cells were re-suspended in 100 μL of PBS 1X. Samples were analyzed within 30 min. Procedures and analysis were conducted in accordance with manufacturer’s recommended procedure with minor modifications.

## 3. Results

### 3.1. Synthesis and Characterization of CaS Nanoclusters

The molecular level heating associated with the use of microwave radiation (MW) offers the unique advantage to stop the decomposition of DMSO and turn “on” and “off” the supply of sulfide necessary for metal–sulfide synthesis [27]. The approach offers the unique advantage to stop the supply of sulfide ions at different stages of the chemical processes allowing to halt the nucleation and growth processes and control cluster or particle size. In the case of CaS, the process can be described according to the following process:

(1) $${\text{nCa}}^{2+}\underset{\text{MW}}{\overset{\text{DMS}}{\to }}{\left(\text{CaS}\right)}_{\text{n}}$$

with n=1 (monomers), 2, (dimers) and so on, until nanostructures are formed. Exposure of diluted solutions of $\text{Ca}{\left({\text{CH}}_{3}{\text{CO}}_{2}\right)}_{2}$ in DMSO to microwave radiation for short periods of time results in the formation of CaS monomers and clusters, as revealed by the lack of absorption bands due to phonon-coupled electronic transitions from the valence to the conduction band. Solutions exposed to microwave heating with initial $\text{Ca}{\left({\text{CH}}_{3}{\text{CO}}_{2}\right)}_{2}$ to DMSO ratios larger than 10^−3^ result in the formation of CaS nanostructures, as evidenced by the well-defined structure that results from the phonon-coupled valence to conduction band electronic transitions [29,30].

**Figure 1.**
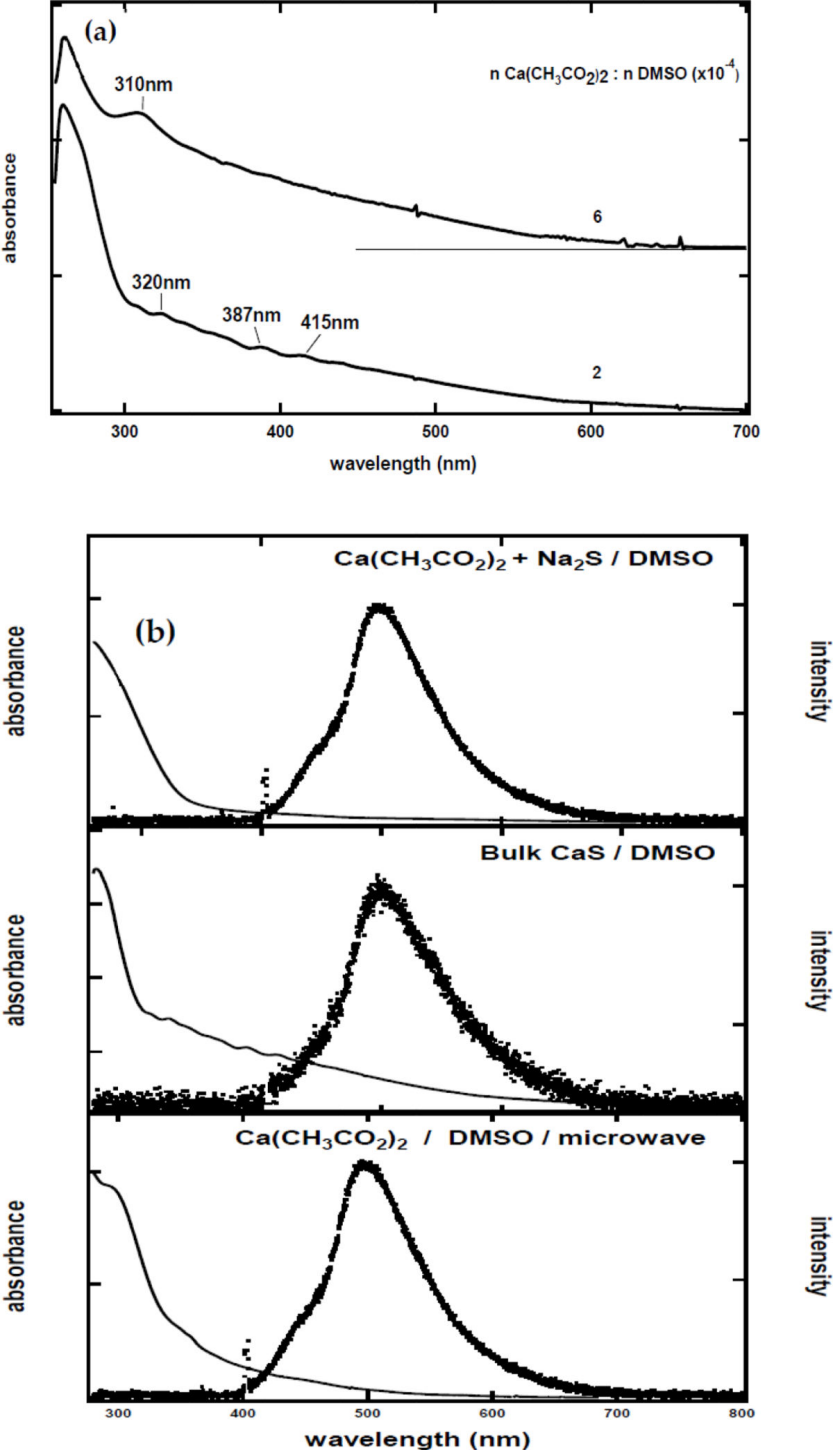
(**a**) UV-visible spectra of CaS nanostructures prepared from the reaction of $\text{Ca}{\left({\text{CH}}_{3}{\text{CO}}_{2}\right)}_{2}$ with DMSO in a microwave. The $\text{Ca}{\left({\text{CH}}_{3}{\text{CO}}_{2}\right)}_{2}$ to DMSO ratios of the spectra are (**a**) 2 × 10^−4^ and (**b**) 6 × 10^−4^. (**b**) Absorption and emission spectra of CaS prepared from the double ion exchange reaction in DMSO (**top**), dissolution of bulk CaS in DMSO (**middle**), and microwave decomposition of $\text{Ca}{\left({\text{CH}}_{3}{\text{CO}}_{2}\right)}_{2}$ in DMSO (**bottom**).

Representative UV visible absorption spectra of the dispersions employed in the experiments described here are displayed in Figure 1. The absorption spectrum of the dispersion with a 2.0 × 10^−4^ ${\text{Ca}}^{2+}$-to-DMSO ratio has a well-defined band below 300 nm and well-defined features at 320, 387, and 415 nm. The absorption spectrum of CaS obtained from the dispersion with a 6.0 × 10^−4^ $\text{Ca}{\left({\text{CH}}_{3}{\text{CO}}_{2}\right)}_{2}$-to-DMSO mole ratio, on the other hand, has a well-defined band that extends to 600 nm. These observations are consistent with the absorption of light by CaS reported in the literature. The optical absorption of CaS monomers in the gas phase has transitions at 300, 500, and 1200 nm, while only transitions in the UV are predicted in dispersions of the monomers in DMSO [29,31,32]. Fluorescence measurements, on the other hand, of these dispersions reveal an emission band centered at 497 nm upon excitation with 405 nm [28,29,33,34]. The emission spectra, illustrated in Figure 1b, are identical to the (a) one obtained when the solutions of Na_2_S and $\text{Ca}{\left({\text{CH}}_{3}{\text{CO}}_{2}\right)}_{2}$ in DMSO are mixed and (b) dispersions obtained by dissolving solid CaS in DMSO. We conclude that the microwave-mediated decomposition of DMSO in the presence of $\text{Ca}{\left({\text{CH}}_{3}{\text{CO}}_{2}\right)}_{2}$ results in the formation of CaS.

**Figure 2.**
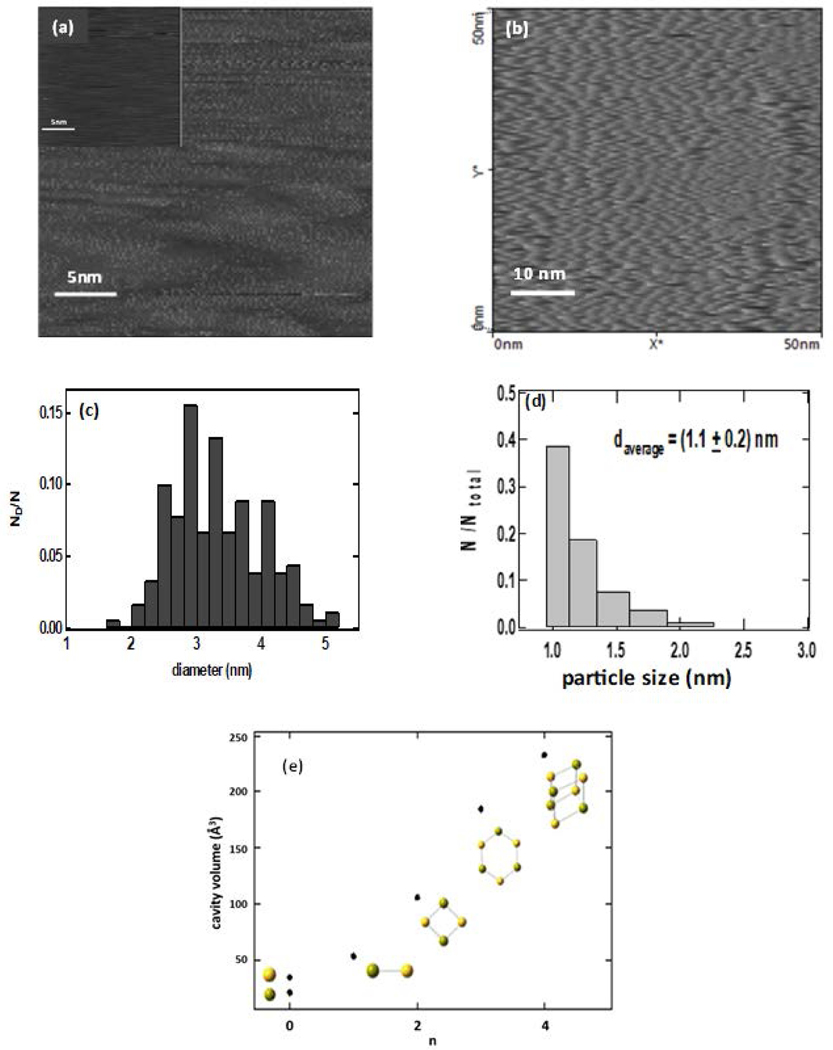
The STM images obtained for dry deposits of CaS with initial $\text{Ca}{\left({\text{CH}}_{3}{\text{CO}}_{2}\right)}_{2}/\text{DMSO}$ ratios of (**a**) 2 × 10^−4^ and (**b**) 6 × 10^−4^. The insert in (**a**) represents the STM of the graphite substrate employed for the measurements. The particle size distribution obtained from the STM and light scattering measurements for dispersion with a $\text{Ca}{\left({\text{CH}}_{3}{\text{CO}}_{2}\right)}_{2}$ to DMSO ratio of 6 × 10^−4^ prepared in the microwave are labeled (**c**,**d**). The calculated volumes of ${\text{Ca}}^{2+}$, ${\text{S}}^{-2}$, and optimized structures of ${\left(CaS\right)}_{n}$ nanoclusters (n=1,2,3, and 4) in DMSO are summarized in (**e**). The calculations were performed at the DFT/B3LYP/DGZVP level of theory.

STM images of dry deposits of these dispersions, as well as particle size distributions obtained from light scattering and imaging measurements, are summarized in Figure 2. The average particle sizes obtained from light scattering measurements of CaS dispersions are summarized in Table 1. The light scattering measurements indicate that CaS nanostructures prepared with the initial $\text{Ca}{\left({\text{CH}}_{3}{\text{CO}}_{2}\right)}_{2}/\text{DMSO}$ 4 × 10^−4^ ratio have a hydrodynamic diameter that is below the detection limit of 0.8 nm of the instrument employed for the measurement [8]. This result is consistent with the STM image presented in Figure 2a, which shows discrete roughness features smaller than 1 nm. The particle size distribution obtained from the STM and light scattering measurements for a dispersion with a $\text{Ca}{\left({\text{CH}}_{3}{\text{CO}}_{2}\right)}_{2}/\text{DMSO}$ ratio of 6 × 10^−4^ are summarized in Figure 2c,d. The STM measurements reveal an average particle diameter of (3.1 ± 0.3) nm, while a hydrodynamic diameter of (1.1 ± 0.2) nm is determined for dispersions with initial $\text{Ca}{\left({\text{CH}}_{3}{\text{CO}}_{2}\right)}_{2}/\text{DMSO}$ ratios of 6 × 10^−4^. The slightly larger average particle size obtained from the STM images likely results from the coalescence of particles in the dry deposits. Since the CaS in DMSO was used for the cancer and normal cell experiments described in the next section, we use the hydrodynamic ratio obtained from the light scattering ratio hereafter.

It is of interest to consider possible CaS structures in the range of a few angstroms to about 1 nm that can account for the observed STM and light scattering measurements. The diameter of optimized clusters (n=1,2,3 and 4) in DMSO can be calculated using density functional theory using the B3LYP functional and the DGZVP basis set. The diameters are obtained from the solvated cluster volume assuming a spherical solvent cage. The predicted diameters of solvated and optimized structures that correspond to the monomer (CaS), dimmer (CaS)_2_, trimer (CaS)_3_, and tetramer (CaS)_4_ are between 0.5 and 0.8 nm, respectively. The solvated diameters of these clusters fall within the range of hydrodynamic diameters determined experimentally in the light scattering measurements. We propose that the CaS structures prepared by the methods described here are clusters of calcium sulfides. These nanoclusters are attractive structures for biomedical applications due to their size that facilitates cellular uptake, access to the smallest vasculature in humans, and elimination by the body as well as optical and chemical properties.

#### 3.1.1. Effect of CaS in Human Carcinoma Cells

**Figure 3.**
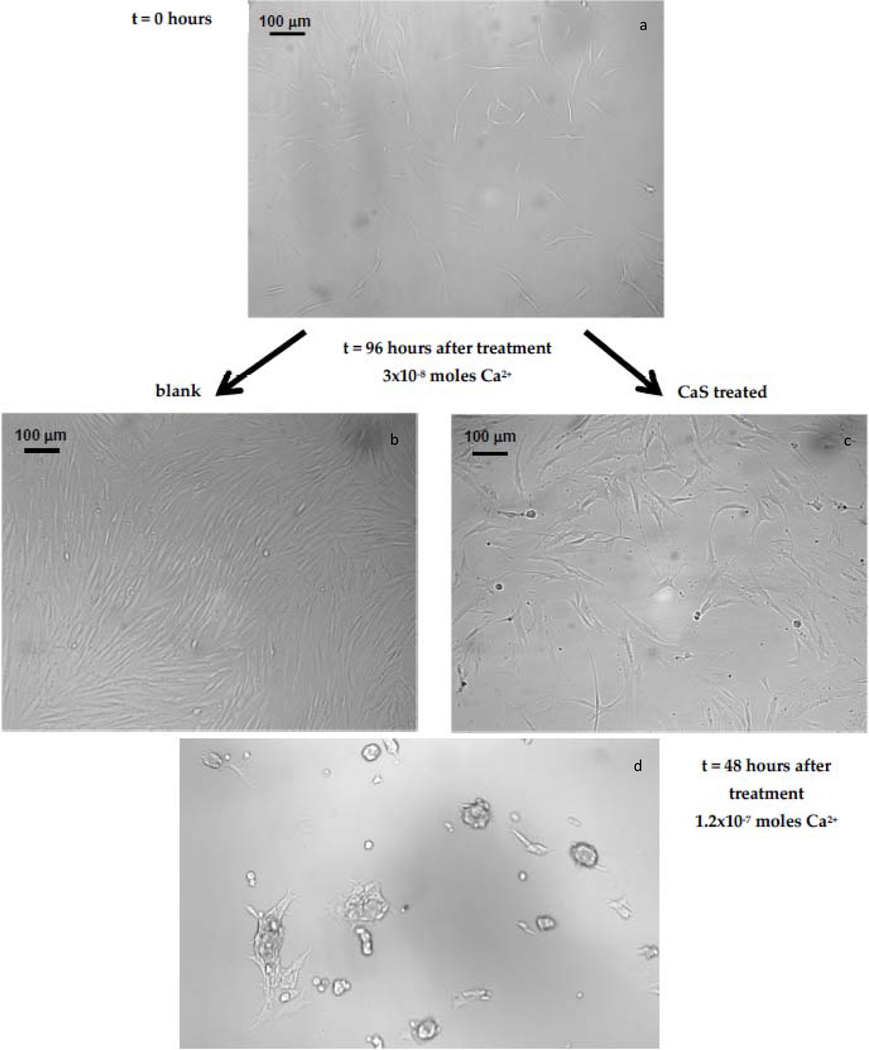
Images of adenocarcinoma cells obtained: (**a**) 24 h after inoculation, (**b**) the control 96 h after preparing the culture, and (**c**) 96 h after adding a single dose of CaS NS. The amount of ${\text{Ca}}^{2+}$ in the NS dispersion is 4 × 10^−8^ moles. (**d**) A representative image of adenocarcinoma cells 48 h after a dose of CaS NS with a ${\text{Ca}}^{2+}$ ion content of 1.2 × 10^−7^ mol is indicated at the bottom of the figure.

Representative images of carcinoma cells obtained 72 h after a single calcium sulfide dose, as well as those not exposed to CaS, are displayed in Figure 3. The total calcium content in the dose was under3 × 10^−8^ moles. The effect of adding CaS to the media revealed a sharp decrease in the density of cells 96 h after the dose, as compared to those cells not exposed to CaS. Images of carcinoma cells at approximately 10 h after a single CaS dose containing a larger calcium content ( 16 × 10^−8^) of mol obtained at a higher magnification are displayed in the bottom of the figure. There was an evident change in the morphology of the elongated fibroblasts. We can identify the presence of blebs and protuberances in many of the cells. These changes in morphology are generally associated with apoptotic events.

#### 3.1.2. Effect of CaS Clusters in Human Carcinoma Cell Density

**Figure 4.**
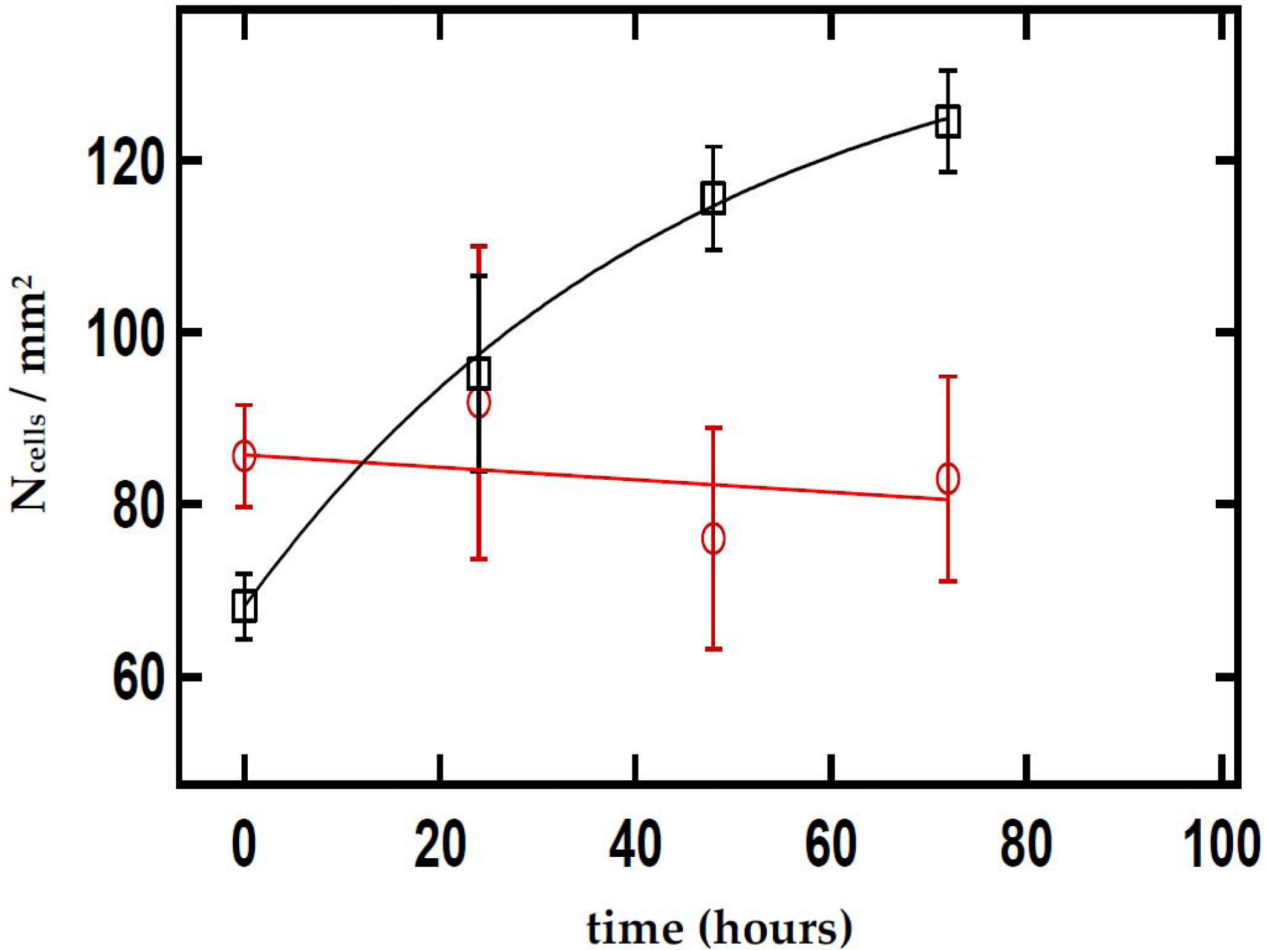
The open squares and circles represent the dependence of carcinoma cell density fed with normal media and media mixed with CaS nanoclusters. The total amount of ${\text{Ca}}^{2+}$ in the nanocluster dispersion is 4 × 10^−8^ moles. The time t=0 h corresponds to the time of the initial dose. The solid black curve and red line are to guide the eyes through the discussion and do not represent a model fit. The spectrum of the dispersion containing the CaS nanoclusters used for the measurements is indicated in the insert at the lower right-hand side of the figure.

We studied the effect of CaS nanoclusters on the growth and survival of carcinoma cancer cell cultures. The effect of a single dose of a dispersion containing CaS clusters on the density of carcinoma cells as a function of time is summarized in Figure 4. The optical absorption spectrum of the CaS dispersion employed is displayed in the insert. The optical absorption spectrum is characterized by bands around 280 nm and a long wavelength tail that extends to 600 nm, consistent with a mixture of monomers and clusters. Carcinoma cells exposed to the CaS dispersion did not exhibit significant growth with time compared to carcinoma cells not exposed to the CaS dispersion. Indeed, there was a monotonic decrease in the density of carcinoma cells exposed to CaS from 90 to less than 80 cells/mm^2^ in the first 72 h following the dose. The density of cells decreased at a rate of (0.10 ± 0.1) cells per mm^2^ per hour. Carcinoma cells not exposed to the CaS dispersion, on the other hand, exhibited a growth from 70 to about 120 cells/mm^2^ in the first 72 h. The rate of increase in the cell density was (48 ± 2) cells per hour per mm^2^. These results led us to conclude that the dose of CaS clusters reduces carcinoma cell proliferation.

#### 3.1.3. Effect of CaS Nanostructures (NS) in Carcinoma Cells

**Figure 5.**
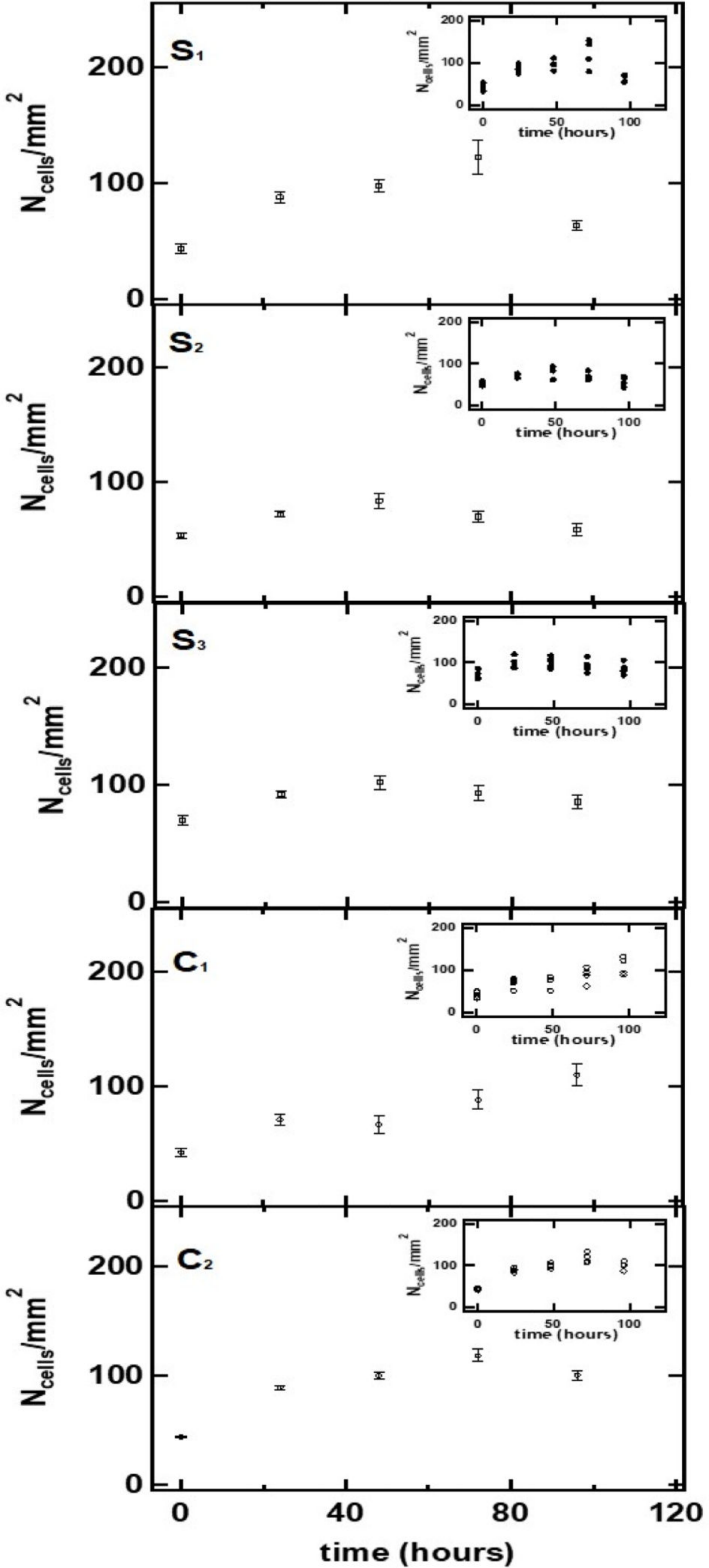
The density of adenocarcinoma cells following a single dose of CaS NS (${\text{S}}_{1}$, ${\text{S}}_{2}$, ${\text{S}}_{3}$) and vehicle controls (${\text{C}}_{1}$ and ${\text{C}}_{2}$). The error bars represent the standard deviation of the measurements. The individual measurements performed are indicated in the insert in the upper right corner of each graph. The amount of ${\text{Ca}}^{2+}$ in the NS dispersion corresponds to 4 × 10^−8^ moles.

The insert in the upper right-hand side of Figure 5 represents the results of the measurements of carcinoma cell density at different times following a single CaS NS dose. The plots labeled S_1_, S_2_, and S_3_ represent the average carcinoma cell density on each sample at the indicated times following the dose of CaS NS in a DMSO dispersion. The results from two independent controls, labeled ${\text{C}}_{1}$ and ${\text{C}}_{2}$, are also included in the figure. One of the control samples, ${\text{C}}_{1}$, was fed with equal volumes of DMSO in the media as the ${\text{S}}_{1}$, ${\text{S}}_{2}$, and ${\text{S}}_{3}$ samples, while the other control sample, ${\text{C}}_{2}$, was fed with normal media. The general trend found was an increase in carcinoma cell density in the first 24 h in all samples. The density of cells started to decrease about 48 h following the CaS dose, while the density of cells in the two controls increased during this period. The average rate of decrease in the cell density of samples exposed to CaS is (0.7 ± 0.2) cells/mm^2^ per hour. We conclude that CaS nanostructures reduce carcinoma cell proliferation. However, they appear to act somewhat slower than the CaS clusters, where the inhibition in cancer cell proliferation was found immediately following the dose.

**Figure 6.**
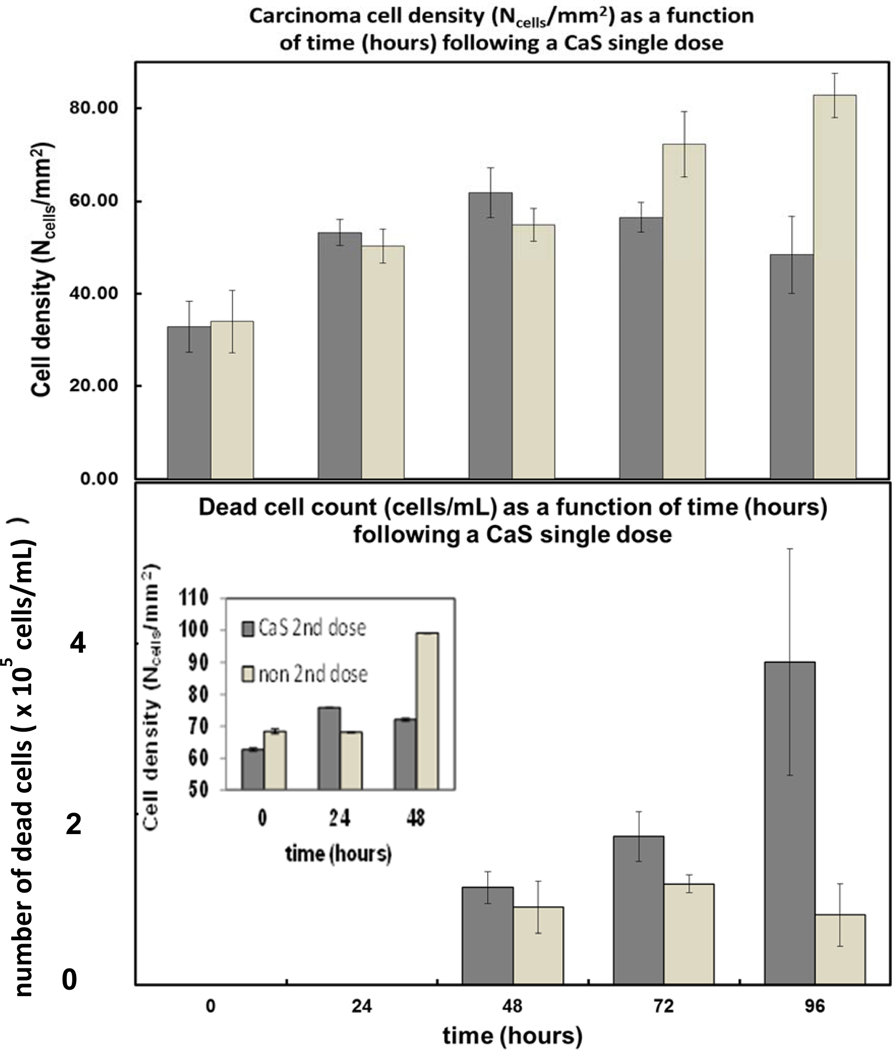
The gray bars in the upper and lower panel summarize the effect of a single dose of CaS NS on the density of carcinoma cells and the number of dead carcinoma cells, respectively, as a function of time. The light gray bars in the upper and lower panel represent the density of adenocarcinoma cells and number of dead carcinoma cells in cell cultures fed with media not containing the CaS nanostructures, respectively. The values reported represent the average of 15 measurements performed on different regions of three different flasks. The error bars represent the standard error of the measurements. The dark and light gray bars in the insert represent the dependence of the density of adenocarcinoma cells exposed to a second dose of the CaS NS and exposed to one dose, respectively, on time. The time t=0 h is taken to correspond to the time of the initial dose. The total mol of ${\text{Ca}}^{2+}$ in the NS dispersion is 4 × 10^−8^.

The gray bars in the upper and lower panels of Figure 6 summarize the effect of a single CaS dose on the average density of carcinoma cells and the number of dead carcinoma cells, respectively, as a function of time. The white bars in the upper and lower panel of Figure 6 represent the average density of cells and the number of dead carcinoma cells in the controls. Each reported value represents the average of the 15 independent measurements performed each day on different regions of samples S_1_, S_2_, and S_3_. The error bars represent the standard error of the measurements.

The average density of carcinoma cells increased monotonically in the first 48 h following the CaS NS dose. The density of cells fed with CaS in the media started to decrease at about 48 h following the CaS dose. The rate of increase of the density of cells in the first 72 h was about (0.4 ± 0.1) (cells/mm^2^) per hour, slightly lower than the rate of growth of carcinoma cells in the control, which was estimated to be (0.50 ± 0.04) cells per mm^2^ per hour in the same time range.

There was a well-defined decrease in the density of cells between 48 and 72 h following the dose. The number of dead cells in this group, on the other hand, increased from 100,000 to 150,000 to over 400,000 in the first 48, 72, and 96 h, respectively, following the single dose of CaS. The rate of increase in the number of dead cells was (3.8 ± 0.8) × 10^3^ cells/h. The marked decrease in the density of cells and sharp increase in the number of dead cells was not observed in the carcinoma cells used as a control. We conclude that CaS induce cell death within 48 h following a single dose.

The density of cells as a function of hours following a second dose of CaS is illustrated in the insert in Figure 6. The gray and light-colored bars represent the density cells exposed to a second dose of CaS and control, respectively. Both cell samples were sub-cultivated from cells exposed to a single CaS dose, as described in the previous paragraphs. Cell growth was followed for a period of 48 h. The number of control cells increased to about 100 cells/mm^2^ in the first 48 h. The number of cells exposed to a second dose did not reach this cell density in this period.

#### 3.1.4. Effect of CaS Nanoclusters on Normal Human Fibroblasts

**Figure 7.**
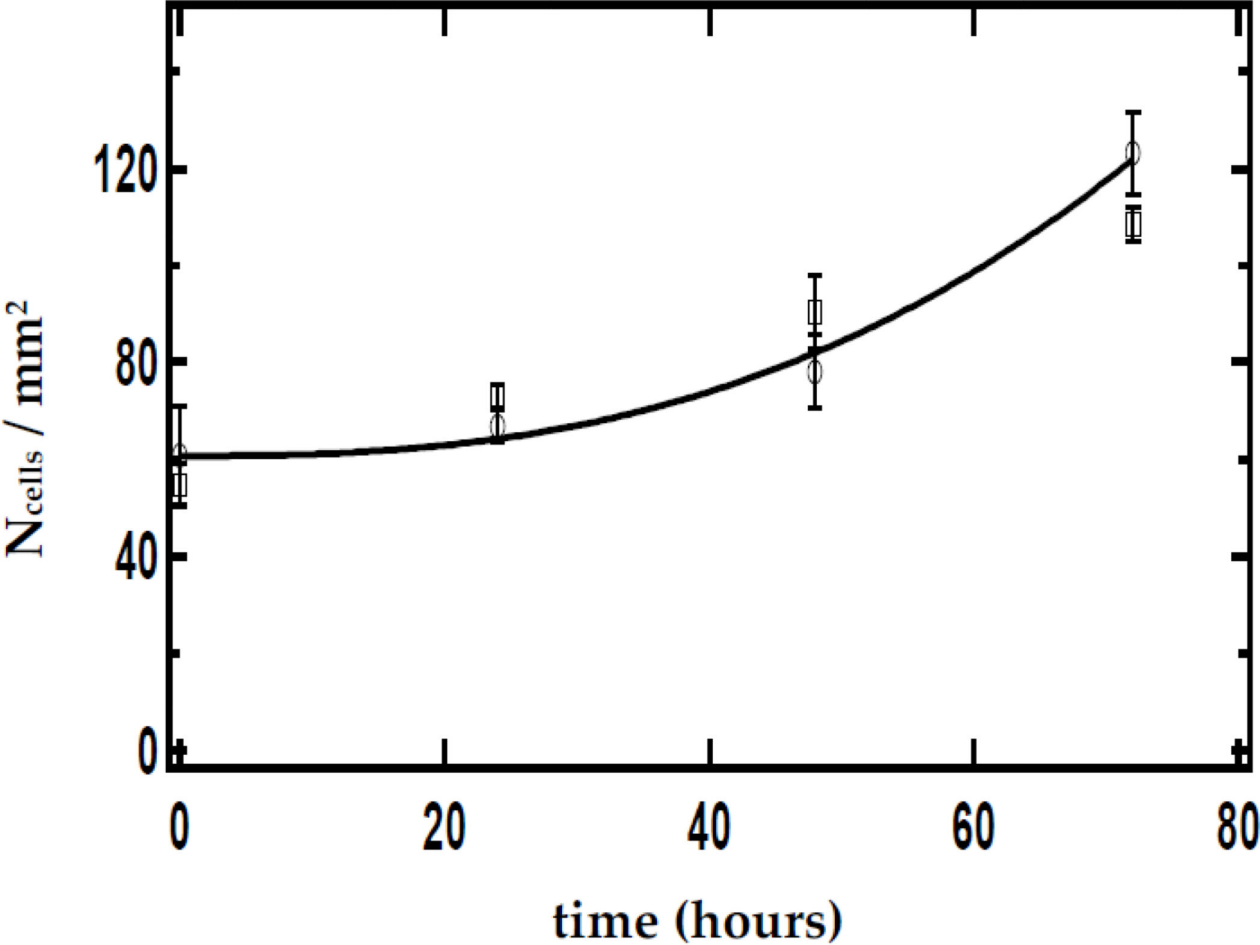
The squares and circles represent the dependence of normal fibroblast cell density fed with normal media and media mixed with CaS clusters, respectively. The time t=0 h is taken to correspond to the time of the initial dose. The solid line is to guide the eyes through the discussion and does not represent a model fit. The spectrum of the CaS nanocluster dispersion employed is indicated in the insert. The total number of mol of ${\text{Ca}}^{2+}$ in the dispersion is 4 × 10^−8^.

The dependence of the density of normal fibroblast exposed with to CaS clusters in the media on time is summarized in Figure 7. A single CaS dose was prepared by adding 200 μL of a CaS dispersion to 12 mL of the media used to feed the cell culture. This corresponds to a total calcium content of the order of 10^−8^ moles. The density of normal fibroblasts exposed to media not containing the CaS dispersion is also indicated in Figure 3b. The density of cells increased with time in both cell cultures. Within the experimental uncertainty, we could not differentiate the rate of increase of cell density in either cell line. The density of cells increased from about 50 to over 110 cells/mm^2^ in a 72 h period. These results suggest that CaS has little effect, if any, on the proliferation of normal fibroblasts.

### 3.2. Effects of CaS Nanoclusters on Cell Cycle Progression

**Figure 8.**
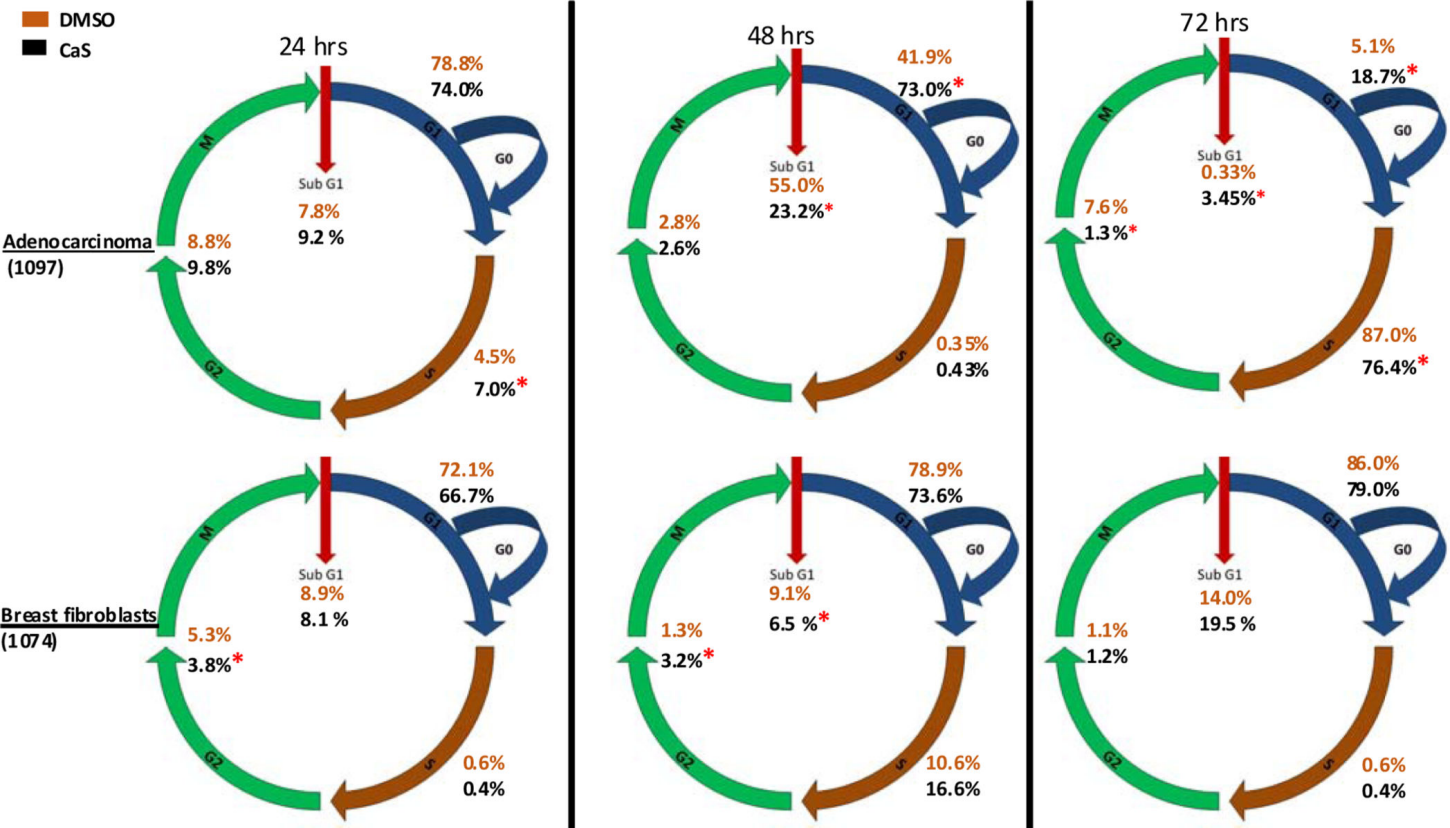
The upper and lower panel summarizes the effect of CaS nanoclusters in the progression of the cell cycle of breast adenocarcinoma and normal fibroblasts, respectively. The vehicle control is 2% DMSO. The total amount of ${\text{Ca}}^{2+}$ in the dispersion is 4 × 10^−8^ moles. The * indicates statistically significant values.

The results of the effect of 500 μL of the CaS nanocluster dispersion in the human carcinoma and normal fibroblasts cell cycle is summarized in the upper and lower panels of Figure 8, respectively. Cell cycles were determined at 24, 48, and 72 h after the cells were exposed to the CaS nanoclusters. DMSO was used as a vehicle control. The percentage of adenocarcinoma cells or normal fibroblasts determined at each phase of the cell cycle is indicated in the figure. Statistically significant differences (p-value < 0.05) in the normal cell population exposed to CaS and treated normally are indicated by a red star. We first focus on the effect of nanoclusters on the cell cycle of benign cells. We did not observe any statistically significant differences in the population of normal cells treated with CaS and the vehicle control in the S or G0/G1 phases. We found a statistically different benign cell population in the G2 phase at 24 and 48 h post-treatment and the Sub G1 phase at 48 h. The population of benign cells treated with CaS nanoclusters was smaller than the vehicle control 1.5% and 2.6% at 24 and 48 h, respectively. The population of normal fibroblasts exposed to the CaS nanocluster dispersion was slightly lower than the corresponding population determined for cells exposed to the vehicle control in the G2/M phase. This initial decrease in the G2/M phase was counteracted at 48 and non-existent at 72 h.

Human carcinoma cells, on the other hand, treated with 500 μL of CaS nanocluster dispersion exhibited a decreased ability to properly enter the cell cycle marked by a decrease in cell concentration in the G0/G1 phase at 24 hours post-treatment and then an increase in cells held in the SubG1 and G0/G1 phases up to 72 h post-treatment. We did not observe any statistically significant differences in the cell population in the S or G2/M phases, suggesting that CaS nanoclusters’ main mode of action in the first 24 and 48 h is centered around early cell cycle checkpoints.

### 3.3. CaS Nanoclusters and Cell Survival

**Figure 9.**
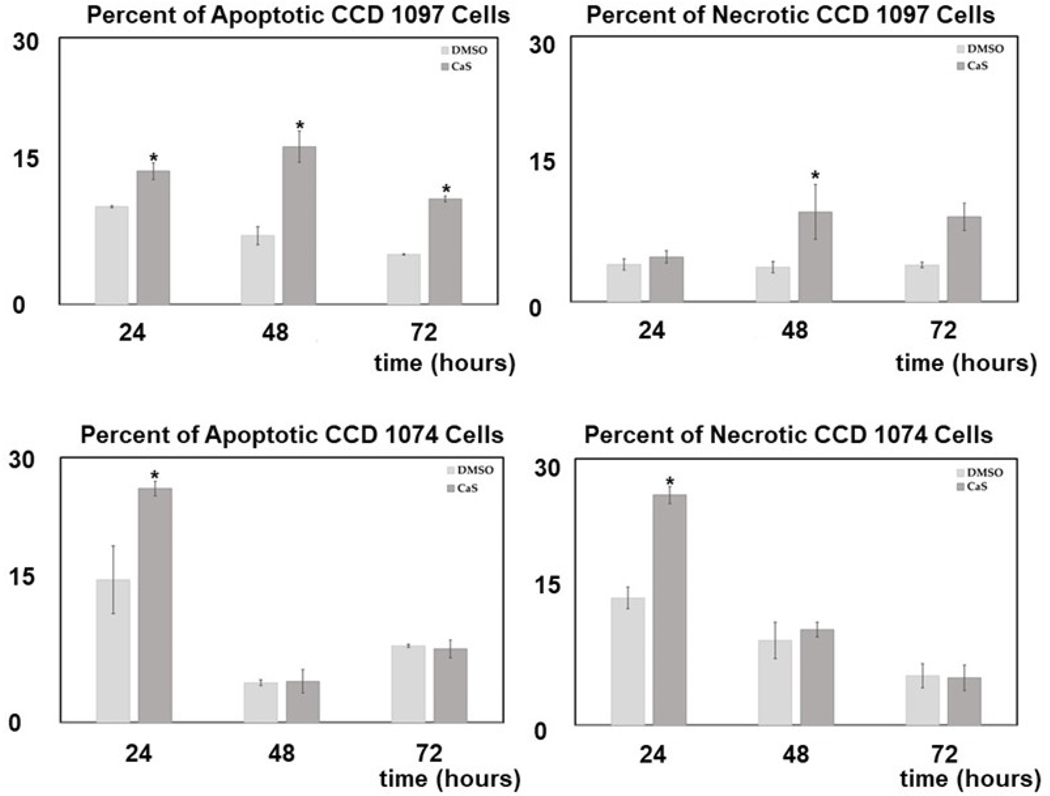
The upper panel summarizes the effect of CaS nanoclusters in the number of apoptotic and necrotic adenocarcinoma cells. The effect of these nanostructures in the number of apoptotic and necrotic normal fibroblasts is indicated in the lower panel. The (*) mark indicates statistically significant values.

The percentage of apoptotic and necrotic human carcinoma and normal fibroblasts exposed to the CaS dispersion and vehicle control are summarized in Figure 9. There was a modest, yet statistically significant increase in the concentration of cells that were stained as apoptotic and necrotic at 24 h post-treatment in our normal fibroblast cells. Exposure of the normal cells to CaS nanoclusters does not result in a significant difference in the percentage of apoptotic or necrotic normal fibroblasts as compared to the vehicle control. In contrast, the carcinoma cell line exhibited statistically significant increases in apoptotic cells that were statistically significant at 24, 48, and 72 h. Similarly, the concentrations of necrotic cells increased from 48 up to 72 h. It is apparent that any effects of CaS on normal fibroblasts were not detrimental to the overall population and were short-lived, while in contrast, CaS nanoclusters affected carcinoma cells in a rapid and long-lasting manner.

### 3.4. Interaction of CaS with Relevant Biological Functional Groups

We performed theoretical calculations at the DFT/B3LYP/6–311G level of theory to learn about favorable interactions of CaS with functional groups relevant to the cell environment. We sought to identify those interactions that could result in the dissociation of CaS. The results of the optimized structures are summarized in Table 2. None of the optimized structures exhibited negative frequencies. The bond length of CaS was found to be 2.58 Å. This distance is short enough to consider localized interactions of CaS with relevant functional groups that may be found in macromolecules useful in the interpretation of results. The optimization process was performed by placing CaS at about 2.7 Å from a given functional group. Two calculations were performed for each functional group interacting with CaS. These calculations differed only in the initial orientation of CaS with respect to the functional group. In one calculation, CaS was oriented with the Ca end pointing toward the functional group of interest. The sulfur end pointed toward the functional group in the second calculation. We found that the optimized structures of these complexes were independent of the initial orientation of CaS with respect to the functional group. The resulting distances between the closest atom in the functional group and CaS in the optimized complex are also indicated in Table 2. These distances ranged from about 2 to 3 Å. All interactions were found to be exergonic. The interaction energies reported in Table 2 are taken as the difference in energy between the optimized complex and the initial CaS and organic molecule. The interaction of CaS was found to interact with the thiol (R-SH), alcoholic (R−OH), and amino ($\text{R}-{\text{NH}}_{2}$) functional groups by the calcium end. The oxygen atoms in the carboxylic acid functional group also exhibited a strong interaction with the calcium end of CaS. The complete proton transfer from the carboxylic acid group to the sulfur end was found only in phenylalanine.

The relevant physiological pH is significantly higher than the pK_a_ of most organic carboxylic acids [35]. Functional groups containing ${\text{NH}}_{4}^{+}$ and ${\text{COO}}^{-}$ were present at pH values between 6 and 7.45, which were typical values found in cancer and normal cells, respectively [36]. The ammonium (${\text{R-NH}}_{3}^{+}$) and carboxylate (${\text{R-COO}}^{-}$) functional groups were found to interact with the sulfur and the calcium end of CaS, respectively. The interaction between the calcium end of CaS and the carboxylate anion resulted in lower energy and shorter distances than the interaction of the sulfur end with the ammonium functional group. None of the interactions studied resulted in the dissociation of Ca-S into calcium and sulfide ions, although we found that the aromatic group in phenylalanine interacted with the CaS monomer well enough to facilitate the proton transfer from the carboxylic group to form ${\text{CaSH}}^{+}$. This proton transfer was not observed with other carboxylic acids, including acetic acid and glycine.

## 4. Discussion

The absorption and emission spectra of dispersions prepared from the decomposition of DMSO in the presence of $\text{Ca}{\left({\text{CH}}_{3}{\text{CO}}_{2}\right)}_{2}$ were remarkably similar to the one obtained for dispersions prepared by dissolving ${\text{CaS}}_{\left(\text{s}\right)}$ mineral in DMSO or from the reaction of ${\text{Na}}_{2}\text{S}$ and $\text{Ca}{\left({\text{CH}}_{3}{\text{CO}}_{2}\right)}_{2}$ in DMSO. These dispersions absorb light from the UV to the visible. The observed emission was consistent with the fluorescence spectra of CaS nanostructures obtained from the microwave-mediated decomposition of other organic sulfides [28].

There are several models to describe cell proliferation discussed in the literature. The application of these models to specific cases depends on the complexity of the cell culture and its environment. One of the simplest mathematical models is a power law of the form:

(2) $$N(t)=N(t=0)*t^{\alpha }$$

where N(t) and N(t=0) represent the number of cells at any time t and the initial number of cells after inoculation has been completed, respectively. In Equation (2), t is time and α is constants. Modeling the experimental data with a power law of the form described by Equation (2) resulted in the values of α summarized in Table 3. The values of α for the growth of carcinoma and normal fibroblasts used as a control in the experiments described in Sections 3.3 and 3.4 were between 0.2 and 0.5 in the region of maximum growth, and between 0.4 and 0.49 in the entire time scale of our measurements. These values were lower than the typical third power law found in tumors, likely the result of two-dimensional growth, but fell within the power measured in several systems [37]. Significantly, the values of α found in the proliferation of carcinoma and normal fibroblasts reflect an increase in cell density with time. The value of α for those carcinoma cells exposed to a single dose of CaS, on the other hand, was negative and reflects a decrease in carcinoma cell density. This marked difference in the proliferation of carcinoma cells and normal fibroblasts led us to propose that CaS is cancer-specific.

The development of cancer-specific chemotherapies has been the subject of basic and applied research for years. Major limitations in the application of nanotechnologies as targeted cell cancer and metastatic therapy include toxicology [38–40], lack of biodegradability [41–44], and size to facilitate access to the—normal and newly developed due to angiogenesis—small vasculature system in humans and elimination by the body [45–49]. The results presented here encourage the use of CaS nanostructures as a target cell cancer and metastasis therapy. The results presented in Figures 4 and 5 indicate that CaS nanostructures are capable of inhibiting the proliferation of cancer cells without affecting the survival and growth rate of normal cells and encourage further research in vivo. Many apoptotic chemical processes are dependent on ${\text{Ca}}^{2+}$ ion and ${\text{H}}_{2}\text{S}$ concentration [49–53]. We hypothesize that the release of calcium and sulfide ions from CaS is favored in the acidic environment found in cancer cells as opposed to the basic media in normal cells. This is further supported by thermodynamic considerations discussed below.

The standard Gibbs free energies of formations $\text{Δ}G_{f}^{0}$, of ${\text{H}}_{2}{\text{S}}_{\left(\text{aq}\right)}$, ${\text{S}}_{\left(\text{aq}\right)}^{-2}$, ${\text{Ca}}_{\left(\text{aq}\right)}^{2+}$, and ${\text{H}}_{\left(\text{aq}\right)}^{+}$ and ${\text{CaS}}_{\left(\text{s}\right)}$, are summarized in Table 4. Gibbs free energies of formation were obtained from the literature [52]. Using these values, we estimated that the dissolution of solid ${\text{CaS}}_{\left(\text{s}\right)}$ in water:

(3) $$\text{Ca}{\text{S}}_{\left(\text{s}\right)}\to \text{C}{\text{a}}_{\left(\text{aq}\right)}^{2+}+{\text{S}}_{\left(\text{aq}\right)}^{-2}$$

is an endergonic reaction with a standard Gibbs free energy $\left(\text{Δ}G_{\text{rxn}}^{0}\right)=9.62\text{kJ}/\text{mol}$ and is not a spontaneous process.

The pH of normal and tumor cells depends on the tissue from where the sample is taken. The extracellular pH (pHe) of normal cells is slightly basic and ranges from 7.35 to about 7.8 [53]. The pKa of ${\text{H}}_{2}\text{S}$ and ${\text{HS}}^{-}$ is 7.04 and 11.96, respectively [52]. Thus, for pH values found in cancer cells, in the range of 6.2 to 7, we expect sulfide to exist predominantly as ${\text{H}}_{2}\text{S}$, while for pH values between 7 and 11.96, we expect ${\text{HS}}^{-}$ to be the dominant sulfide-containing species. The reaction:

(4) $$\text{Ca}{\text{S}}_{\left(\text{s}\right)}+2{\text{H}}_{\left(\text{aq}\right)}^{+}\to \text{C}{\text{a}}_{\left(\text{aq}\right)}^{2+}+{\text{H}}_{2}{\text{S}}_{\left(\text{g}\right)}$$

is exergonic with a $\text{Δ}G_{\text{rxn}}^{0}\text{of}-109.7\text{kJ}/\text{mol}$ and an equilibrium constant of the order of 10^19^. In Equation (4), we have considered the ${\text{H}}_{2}\text{S}$ formed in the gas phase. The standard Gibbs free energy for the reaction is −103.58 kJ/mol with an equilibrium constant of the order of 10^18^, if the ${\text{H}}_{2}\text{S}$ is formed in an aqueous solution. The sign and magnitude of $\text{Δ}G_{\text{rxn}}^{0}$ for this reaction, either with ${\text{H}}_{2}\text{S}$ formed in the gas or aqueous phases, indicates that the formation of free calcium ions and hydrogen sulfide from CaS is a spontaneous process that releases a significant amount of energy in acidic media. Thus, the formation of ${\text{Ca}}^{2+}$ and ${\text{H}}_{2}\text{S}$ is thermodynamically favored in the acidic pH found in the extracellular fluid of cancer cells but is limited in the basic extracellular environment of benign cells. We propose that the difference in pH results in the selectivity of the CaS nanostructures to limit the growth and proliferation of breast cancer cells with little effect on the corresponding benign cells. Calcium ion signaling in the cytosol can be grouped by their general mechanisms of movement: (1) ${\text{Ca}}^{+2}$ import into the cytosol (influx), (2) ${\text{Ca}}^{+2}$ export from the (sequestering), and (3) ${\text{Ca}}^{+2}$ buffering. Small transient events of ${\text{Ca}}^{+2}$ movement (nM range) modulate cell proliferation and apoptosis [54].

In general, cells spend most of their time in the G1 and S phases of the cell cycle due to the time needed to prepare the machinery and replication of DNA. Most antineoplastic agents target the cell cycle as a means to halt cancer. Drugs such as Etoposide, a topoisomerase-2 complex poison used to treat lung cancer, prevent cancer cells from completing the S phase by binding to the topoisomerase-2/DNA complex [55]. However, our data suggest that CaS nanoclusters affect early cell cycle stages SubG1 and subsequently G1/G0 of cancer cells, preventing the cancer cells to enter the S phase. G1/G0 is recognized as the most variable of the phases because it is here where cells can withdraw from the cycle [56]. It remains to be elucidated which molecular mechanisms are being activated or deactivated to result in the observed effects. Based on mathematical modeling and the current literature, our CaS nanoclusters, which were selectively up taken by malignant cells, once in the cytosol could disrupt ${\text{Ca}}^{2+}$ transients. While the absence of ${\text{Ca}}^{2+}$ influences cell cycle progression toward senescence, it is the ${\text{Ca}}^{2+}$ transients, successive events of release/sequestering of ${\text{Ca}}^{2+}$, and the patterns of these events that facilitates the regulation of cell cycle checkpoints. These waves of cytosolic ${\text{Ca}}^{2+}$ can originate from extracellular or intracellular sources. We believe this to be the case as we observed changes in cell cycle progression of malignant cell when treated with CaS nanoclusters [57].

Our calculation demonstrates that CaS is a spontaneous generator of ${\text{S}}^{2-}$ which in acidic environments favors the production of hydrogen sulfide (${\text{H}}_{2}\text{S}$) over the ${\text{HS}}^{-}$ ion. ${\text{H}}_{2}\text{S}$ is an endogenously generated gaseous transmitter that studies have shown induces pro-survival effects, and it is also a reactive oxygen species (ROS) and oxidative stress generator [58]. Oxidative stress is not easily overcome and can have a snowball effect. To manage oxidative stress, the cell must invest in specialized enzymes, and—with a diverted metabolism—cancer cells are more prone to suffering oxidative stress. DNA damage, destruction of proteins and lipid membrane ER stress, and ultimately cell death are the results of unmanaged oxidative stress. CaS nanoclusters induced apoptosis from 24 to 72 h in the malignant cells. This led us to understand that although the decomposition of CaS nanoclusters is thermodynamically spontaneous, the effects of this decomposition are long-lasting. Biochemical processes involving sulfides and calcium may account for the programmed cell death observed here. They can work independently or, together by different mechanisms. The investigation of the cellular and molecular mechanisms underlining the physiological roles of calcium and sulfide ion-dependent self-signaling has been clearly demonstrated in many cell types, including neurons, cardiomyocytes, and endothelial cells, and has also been associated with relevant biological processes such as cardiac contraction, inflammation, sensory transduction, and angiogenesis. The most striking feature of this relationship is the ability of sulfide to either inhibit or activate ${\text{Ca}}^{2+}$ entry depending on the molecular nature of the ${\text{Ca}}^{2+}$ entry pathway [59–66]. In particular, we note that patients treated with calcium channel blockers or hypertensive drugs are found to have a higher risk of developing cancer [59]. Further speculation is unwarranted until experiments directed to establish the cellular chemistry of CaS nanostructures are performed.

## 5. Conclusions

In conclusion, the effect of CaS nanostructures—prepared from the microwave-mediated decomposition of dimethyl sulfoxide (DMSO) in the presence of calcium acetate $\text{Ca}{\left({\text{CH}}_{3}{\text{CO}}_{2}\right)}_{2}$—in the proliferation, cell cycle, and death mechanism of adenocarcinoma cells was investigated. Light scattering measurements revealed that dispersions with CaS nanostructures in the size range of a few Å to about 1 nanometer were formed when DMSO was decomposed in the presence of $\text{Ca}{\left({\text{CH}}_{3}{\text{CO}}_{2}\right)}_{2}$. Theoretical calculations at the DFT/B3LYP/DGDZVP level of theory were consistent with ${\left(\text{CaS}\right)}_{n}$ clusters (n=1,2,3, and 4) in DMSO in the size range of 0.4 to 1.1 nm. The absorption spectra of the CaS nanostructures were dominated by strong bands in the UV as well as weaker absorption bands in the visible. We found that a single dose of CaS nanoclusters smaller than 0.8 nm in diameter did not affect the survival and growth of normal fibroblasts, but it inhibited the proliferation of carcinoma cells in vitro. Larger CaS nanoclusters, about (1.1 + 0.2) nm in diameter, have a similar effect on carcinoma cell proliferation and survival rate. Human carcinoma cells treated with CaS nanocluster dispersion exhibited a decreased ability to properly enter the cell cycle marked by a decrease in cell concentration in G0/G1 phase at 24 h post-treatment and then an increase in cells held in the SubG1 and G0/G1 phases up to 72 h post-treatment. Apoptosis and necrotic channels were found to play significant roles in the death of human carcinoma cells exposed to the CaS nanoclusters. In contrast, any effect on normal fibroblasts appeared to be short-lived and non-detrimental. The interaction between CaS and several functional groups was further investigated by theoretical calculations to learn about the possible interactions that could result in the release of ${\text{Ca}}^{2+}$ or ${\text{S}}^{-2}$ ions. CaS is predicted by theoretical calculations at the DFT/B3LYP/6–311G level of theory to interact with the thiol (R-SH), hydroxide (R−OH), amino ($\text{R}-{\text{NH}}_{2}$), carboxylic acid (R−COOH), ammonium (${\text{R-NH}}_{3}^{+}$), and carboxylate (${\text{R-COO}}^{-}$) functional groups. None of these interactions are predicted to result in the dissociation of CaS. Thermodynamic considerations, on the other hand, are consistent with the dissociation of CaS into ${\text{Ca}}^{2+}$ ions and ${\text{H}}_{2}\text{S}$ in acidic media, both of which are known to cause apoptosis or programmed cell death. Passive uptake and the extracellular pH of carcinoma cells are proposed to result in the selectivity of CaS to inhibit human carcinoma cell proliferation with no effect on normal fibroblast cells. The results encourage further research with other cell lines in vitro as well as in vivo to translate this nanotechnology into clinical use.

## 6. Summary

In summary, we showed that CaS clusters and/or particles affect the proliferation and survival rate of human carcinoma cancer cells in vitro. The CaS clusters appear to be more effective than the CaS NS. Human carcinoma cells treated with CaS nanocluster dispersions exhibited a decreased ability to properly enter the cell cycle marked by a decrease in cell concentration in G0/G1 phase at 24 h post-treatment and then an increase in cells held in the SubG1 and G0/G1 phases up to 72 h post-treatment. Apoptosis and necrotic channels were found to play significant roles in the death of human carcinoma cells exposed to the CaS nanoclusters. In contrast, any effect of the CaS clusters on normal human fibroblasts appeared to be short-lived and non-detrimental. The difference in pH between human cancer and normal fibroblast is proposed to result in the formation of calcium and sulfides, both of which are known to affect the cell cycle. The results encourage further experiments in vivo.

## Figures and Tables

**Table 1.** Particle size diameters obtained from light scattering measurements of dispersions prepared in with microwave radiation. The initial $\text{Ca}{\left({\text{CH}}_{3}{\text{CO}}_{2}\right)}_{2}$ to DMSO concentration ratios of 4 and 6 × 10^−4^.

$\text{Ca}{\left({\text{CH}}_{3}{\text{CO}}_{2}\right)}_{2}/\text{DMSO}$ × 10^−4^ ratio:	(d±σ) nm
4	smaller than 0.8 nm
6	(1.1 ± 0.2)

**Table 2.** Optimized structures and interaction energies of CaS with several functional groups of interest in a cell. The smallest distance between calcium sulfide and the functional group as well as the Ca-S bond length are also indicated in the table.

System	Interaction Energy (kJ/mol)	Cas —Functional Group Minimum Distance (Å)	Ca-S Bond Length (Å)
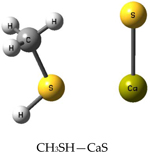	−54.24167478	3.07175	2.59163
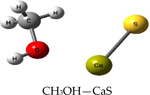	−122.8815391	2.31069	2.59771
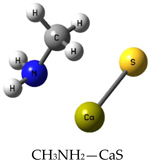	−120.4922772	2.47496	2.59496
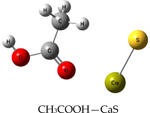	−133.5407041	2.28822	2.60250
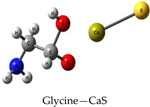	−415.7639143	2.25230	2.80005
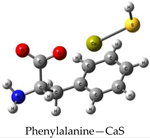	−368.7580388	carboxylic acid: 2.10608 aromatic ring: 3.17687	2.72777
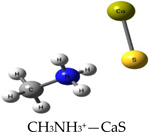	−199.9517525	3.00130	2.54327
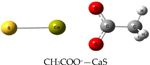	−399.4134555	2.42681	2.65120

**Table 3.** Values of α obtained from a linear regression analysis to a log–log plot according to a power function model for cell growth as a function of time in the region of maximum growth. Values of α that correspond to the total time scale studied are indicated in parenthesis.

Sample	$\ln[N(t=0)\pm \sigma_{N}$	$\alpha \pm \sigma_{\alpha }$
Carcinoma treated	4.8 ± 0.6 (4.6 ± 0.3)	−0.06 ± 0.08 (−0.09 ± 0.1)
Carcinoma untreated	2.694 ± 0.002 (2.8 ± 0.1)	0.4808 ± 0.000 (0.45 ± 0.03)
Fibroblast treated	3.4 ± 0.2 (2.6 ± 0.8)	0.22 ± 0.07 (0.4 ± 0.2)
Fibroblast untreated	2.4 ± 0.4 (2.4 ± 0.3)	0.6 ± 0.1 (0.49 ± 0.09)

**Table 4.** The standard Gibbs free energy ($\Delta G_{f}^{0}$) of formation of probable species containing calcium and sulfide in an aqueous solution.

Species	$\Delta G_{f}^{0}$ (kJ/mol)
${\text{Ca}}^{2+}$	−553.6
${\text{S}}^{2-}$	85.8
${\text{H}}^{+}$	0
CaS	−477.4
${\text{H}}_{2}\text{S}$	−33.6
**Thermodynamics in water ($\Delta G_{f}$ (kJ/mol))**	
${\text{CaS}}_{\left(\text{aq}\right)}\to {\text{Ca}}_{\left(\text{aq}\right)}^{2+}+{\text{S}}_{\left(\text{aq}\right)}^{2-}$	9.62
${\text{CaS}}_{\left(\text{s}\right)}+2{\text{H}}^{+}\to {\text{Ca}}_{\left(\text{aq}\right)}^{2+}+{\text{H}}_{2}{\text{S}}_{\left(\text{g}\right)}$	−109.74

## Data Availability

Not applicable.
